# Existing evidence on the effects of climate variability and climate change on ungulates in North America: a systematic map

**DOI:** 10.1186/s13750-024-00331-8

**Published:** 2024-04-04

**Authors:** Katherine C. Malpeli, Sarah C. Endyke, Sarah R. Weiskopf, Laura M. Thompson, Ciara G. Johnson, Katherine A. Kurth, Maxfield A. Carlin

**Affiliations:** 1 U.S. Geological Survey, National Climate Adaptation Science Center, Reston, USA; 2 Appalachian Laboratory, University of Maryland Center for Environmental Science, College Park, USA; 3 School of Natural Resources, University of Tennessee, Knoxville, USA; 4 Department of Environmental Science & Policy, George Mason University, Fairfax, USA

**Keywords:** Climate effects, Global change, Ungulate ecology, Ungulate management, Weather

## Abstract

**Background:**

Climate is an important driver of ungulate life-histories, population dynamics, and migratory behaviors. Climate conditions can directly impact ungulates via changes in the costs of thermoregulation and locomotion, or indirectly, via changes in habitat and forage availability, predation, and species interactions. Many studies have documented the effects of climate variability and climate change on North America’s ungulates, recording impacts to population demographics, physiology, foraging behavior, migratory patterns, and more. However, ungulate responses are not uniform and vary by species and geography. Here, we present a systematic map describing the abundance and distribution of evidence on the effects of climate variability and climate change on native ungulates in North America.

**Methods:**

We searched for all evidence documenting or projecting how climate variability and climate change affect the 15 ungulate species native to the U.S., Canada, Mexico, and Greenland. We searched Web of Science, Scopus, and the websites of 62 wildlife management agencies to identify relevant academic and grey literature. We screened English-language documents for inclusion at both the title and abstract and full-text levels. Data from all articles that passed full-text review were extracted and coded in a database. We identified knowledge clusters and gaps related to the species, locations, climate variables, and outcome variables measured in the literature.

**Review findings:**

We identified a total of 674 relevant articles published from 1947 until September 2020. Caribou (*Rangifer tarandus*), elk (*Cervus canadensis*), and white-tailed deer (*Odocoileus virginianus*) were the most frequently studied species. Geographically, more research has been conducted in the western U.S. and western Canada, though a notable concentration of research is also located in the Great Lakes region. Nearly 75% more articles examined the effects of precipitation on ungulates compared to temperature, with variables related to snow being the most commonly measured climate variables. Most studies examined the effects of climate on ungulate population demographics, habitat and forage, and physiology and condition, with far fewer examining the effects on disturbances, migratory behavior, and seasonal range and corridor habitat.

**Conclusions:**

The effects of climate change, and its interactions with stressors such as land-use change, predation, and disease, is of increasing concern to wildlife managers. With its broad scope, this systematic map can help ungulate managers identify relevant climate impacts and prepare for future changes to the populations they manage. Decisions regarding population control measures, supplemental feeding, translocation, and the application of habitat treatments are just some of the management decisions that can be informed by an improved understanding of climate impacts. This systematic map also identified several gaps in the literature that would benefit from additional research, including climate effects on ungulate migratory patterns, on species that are relatively understudied yet known to be sensitive to changes in climate, such as pronghorn (*Antilocapra americana*) and mountain goats (*Oreamnos americanus*), and on ungulates in the eastern U.S. and Mexico.

**Supplementary Information:**

The online version contains supplementary material available at 10.1186/s13750-024-00331-8.

## Background

Native ungulate species occupy a diversity of habitats across North America, from Canada’s high arctic to the deserts of Mexico [1]. Through their herbivory, wild ungulates play an important ecological role, regulating processes such as nutrient cycling in temperate forests and Arctic ecosystems [2, 3] and plant productivity and habitat heterogeneity in grasslands [4, 5]. However, ungulate management in North America is challenged by a multitude of anthropogenic and environmental threats that are impacting individuals, populations, and the ability of ungulates to move across the landscape [6]. Changes in habitat [7], physical barriers to movement [8], climate conditions [9, 10], disease transmission [11], and predator communities [9] are of increasing concern to ungulate managers [12]. Of these, an improved understanding of the effects of changing climate conditions has been highlighted as a key information need [13–15]. Climate is an important driver of ungulate life-history characteristics, population dynamics, and migratory behaviors, and changes in climate can directly and indirectly affect the growth, development, fecundity, dispersal, demographic trends, and long-term viability of populations [10, 15] as well as the timing and locations of migratory movements [16, 17]. Understanding the impacts of both short- and long-term changes in climate will provide valuable information to wildlife and land managers. Here, we use the term “climate variability” to refer to interannual or interdecadal fluctuations in temperature and precipitation (e.g., weather), and the term “climate change” to refer to persistent, multidecadal deviations from long-term averages [18].

Many studies have documented the effects of climate variability and climate change on North American ungulates. Climate conditions can directly impact ungulates via changes in the costs of thermoregulation and locomotion [19], with implications for population demographics. For example, overwinter survival of mountain goats (*Oreamnos americanus*) in coastal Alaska is negatively related to higher temperatures during the previous summer, due in part to the increased physiological demands of heat stress that reduce energetic reserves [20]. Precipitation and temperature can also indirectly affect ungulate life-histories and population dynamics through their effects on forage quantity, quality, and accessibility [21–23]. For example, a die-off of barren-ground caribou (*Rangifer tarandus groenlandicus*) in Nunavut, Canada, was linked to abnormally dense snow conditions that restricted the animals from accessing forage [24]. Similarly, following record snowfall in Alberta, malnutrition resulted in the mortality of nearly half of a pronghorn (*Antilocapra americana*) population [25]. In the Southwest U.S., summer drought increased mortality among Sonoran pronghorn (*A. a. sonoriensis*), likely due to a scarcity of nutritional forage [26]. Changes in climate that influence the timing and length of the spring and autumn growing seasons also influence the nutritional condition and demography of ungulates. For example, in Idaho, a longer autumn growing season was found to increase mule deer (*Odocoileus hemionus*) fawn overwinter survival [27].

Forage conditions also drive the movements of migratory ungulate populations in North America [28], and a burgeoning body of literature is bringing to light the effects of climate change on migration [29]. Researchers have found that elk (*Cervus canadensis*) in the Greater Yellowstone Ecosystem delayed departure from winter range habitat when spring green-up occurred later [17], mule deer in the Sierra Nevada migrated earlier in years with earlier green-up and low snow depth [30], and mule deer in western Wyoming experienced decreased foraging benefits from migration during drought years [16]. In addition to changes in forage, climate-driven changes in land cover impact migration. For example, caribou in the Arctic and subarctic are facing new barriers to movement as freshwater lakes and rivers freeze later in the year and thaw earlier [31], forcing caribou to extend their distance traveled to circumnavigate unfrozen water bodies [32]. In extreme cases, mass mortality events occur when animals fall through thin ice [33].

Climate change is also altering trophic cascades and species interactions, with consequences for ungulates. For example, climate conditions can affect predation on ungulates by altering ungulate nutritional condition or mediating the behavior or size of predator populations [34]. In northwestern Montana and southeastern British Columbia, gray wolf (*Canis lupus*) search distance for white-tailed deer (*Odocoileus virginianus*) decreased during deeper snow years, likely due to a combination of reduced mobility and poorer condition of deer [35]. In Isle Royale, Michigan, wolves hunted in larger packs and tripled the number of moose (*Alces alces*) killed per day during snowier years, with negative implications for moose abundance [36]. Climate change can also impact parasitic and viral disease transmission. The emergence of the lungworm nematode *Umingmakstrongylus pallikuukensis* in muskoxen (*Ovibos moschatus*) in the Canadian Arctic and subarctic may be linked to the region’s unprecedented warming trend [37]. Meanwhile, hemorrhagic disease, which affects wild and domestic ruminants, is positively correlated with drought severity in parts of the U.S. [38].

While there is abundant evidence that climate variability and climate change influence ungulates, the responses are not uniform and are likely mediated by local processes and species-specific traits [39]. Furthermore, the evidence is not equally distributed among species and geographies. Following the protocol in Malpeli et al. [40], we carried out a systematic mapping exercise to identify the distribution and abundance of the evidence on how climate variability and climate change affect native ungulates in North America. Cataloguing the existing science on this topic facilitates the identification of the range of climate-related impacts across ungulate species, populations, and geographies, and highlights knowledge clusters and gaps. Additionally, this map helps to address a commonly cited challenge to wildlife management decision making: a lack of time [41, 42]. By removing the time-intensive step of searching the literature, this map enables managers to efficiently identify articles that are relevant to their focal populations and topics. The resulting systematic map can be used by managers to anticipate future changes in ungulate populations as the climate continues to change and can inform future research efforts aimed at enhancing this body of knowledge.

### Stakeholder engagement

We began our process of identifying stakeholder needs by reviewing a series of state wildlife management agency plans that outline key threats and priority research areas related to ungulate management in the U.S. These plans were developed by 11 western U.S. states in 2018, following the signing of Secretarial Order 3362, “Improving Habitat Quality in Western Big-Game Winter Range and Migration Corridors” [43]. This order, which focused on elk, mule deer, and pronghorn, directs the U.S. Department of the Interior’s land management bureaus to work in partnership with designated state wildlife agencies to improve ungulate winter range and migration corridor habitats. As part of this effort, each participating state developed a State Action Plan outlining major threats and priorities related to ungulate migration corridors and winter range habitat. In addition to commonly cited challenges such as wildlife-vehicle collisions and physical barriers to movement such as fences, many plans listed drought, wildfire, disease, and habitat conversion due to the spread of invasive species as key threats to ungulates. They also outlined a clear need for information that will enhance the understanding and protection of ungulate migration routes and seasonal ranges.

In addition to reviewing each State Action Plan, we contacted big game and habitat managers from several U.S. state wildlife management agencies to better understand their priorities and information needs related to ungulate management. As part of these discussions, we inquired about their current understanding of how climate variability and climate change affect the ungulate populations they manage, and the types of information on this topic that support management planning. We also spoke to federal scientists to identify relevant ongoing science activities and to solicit input on the science needs related to ungulates and climate change. The results of this stakeholder engagement and needs assessment process, summarized in Malpeli et al. [44], led to the initial conceptualization of this study. Because our map was not limited to particular ungulate species or outcomes, we extended the geographic scope of this systematic map to encompass all of North America, increasing its relevance to a broader audience.

### Objective of the review

The main objective of this systematic map was to describe the abundance and distribution of the evidence relating to the impacts of climate variability and climate change on the 15 ungulate species of the Order Artiodactyla native to North America. Our primary research question was: *What evidence exists on the effects of climate variability and climate change on ungulates in North America?* This research question was broken into the following Population, Exposure, Comparator, Outcome (PECO) elements, which are further detailed in the “Eligibility criteria” section:

- Population: All subspecies and populations of wild ungulates native to and currently residing in the U.S., Canada, Mexico, or Greenland.
- Exposure: “Direct climate variables” (i.e., temperature, precipitation, climate indices) and their derivatives (e.g., winter severity, drought); climate-attributed changes in "secondary climate variables” (e.g., plant phenology, forage quality and quantity, wildfire, invasive species, disease, predation).
- Comparator: A comparison of at least two different time points, over which there is a quantified, inferred, or projected change in an exposure variable.
- Outcome: Effect on individual life-history characteristics, population dynamics, migratory behavior, or the spatial location or quality of migration corridor or seasonal habitat.

## Methods

Detailed documentation of our systematic map methods is available in Malpeli et al. [40]. Below, we provide an updated description of our methods and describe deviations from the original protocol. The methods used to produce this systematic map follow the Collaboration for Environmental Evidence Guidelines and Standards for Evidence Synthesis in Environmental Management [45] and conform to the ROSES reporting standards (Additional file 1) [46].

### Deviations from the protocol

Several minor deviations from the original protocol were made that focused on adding specificity to our PECO framework. First, we added a temporal specification restricting our map to only include studies focused on “modern climate change”. We used the start of the Industrial Revolution (1760) as our beginning point for modern era climate change. Additionally, several specifications were added to the population component of our framework. First, the original protocol states that ungulates in the U.S., Canada, and Mexico will be included in the map. We updated the geographic scope to include studies located in Greenland. Although politically part of the Kingdom of Denmark, Greenland is geographically part of the continent of North America. Second, we clarified that we considered re-introduced ungulate populations to be native, while we considered introduced populations to be non-native. We also expanded our definition of “wild” ungulate populations and included studies that examined supplementally fed populations (unless the outcome was related to diet or foraging behavior), fenced-in populations (unless the outcome was related to movement behaviors), and semi-domesticated reindeer.

Regarding our exposure variables, we expanded our list of variables to include insect harassment, sea ice, freshwater ice, parasites, harvest, wildlife-vehicle collisions, forage quality, forage quantity, habitat, diet, and resource competition. These variables fall within our “secondary climate variable” category, or variables that were only included when they were described within articles as being linked to changes in direct climate variables (temperature, precipitation, climate indices). During data entry, some of our secondary variables could be entered as either a secondary variable or as an outcome variable, depending on whether the paper quantified the impacts of changes in the exposure variable on ungulates. For example, predation was entered as a secondary variable if a study examined how changes in climate (e.g., snow depth) impacted predation on ungulates, with a measured outcome such as ungulate mortality. Alternatively, predation was entered as an outcome variable if the study examined how climate impacted predation, with predation being the endpoint and no subsequent outcome was measured.

Lastly, we altered the initial proposed structure for recording outcome variables. Instead of structuring our outcome variables within the three originally proposed categories of individual life-history characteristics, population dynamics, and migration, we instead utilized six outcome categories: physiology and condition, habitat and forage, population demographics, disturbances, migratory behavior, and seasonal range and corridor habitat. Many variables that are considered to be life-history characteristics also influence population demographics, therefore we decided to eliminate confusion in categorizing these variables by using a slightly more detailed outcome category structure.

### Search for articles

We conducted a systematic search for academic articles and grey literature using two bibliographic databases and the websites of relevant organizations. We ran searches in September 2020. All searches were conducted in English, due to limitations within the author team, and only English-language publications were included. Additionally, only articles that were available in a digital format were included in the map.

#### Search terms and string

The final search string, used in Web of Science and Scopus, was as follows: *TS* = (("mule deer" OR “black-tailed deer” OR “Odocoileus hemionus” OR "white-tailed deer" OR “whitetail*” OR “Odocoileus virginianus” OR "elk" OR “wapiti” OR “Cervus canadensis” OR "pronghorn" OR “antelope” OR “Antilocapra americana” OR "bighorn sheep" OR “mountain sheep” OR “Ovis canadensis” OR "moose" OR “Alces alces” OR “bison” OR “Bison bison” OR “dall sheep” OR “dall’s sheep” OR “thinhorn sheep” OR “Ovis dalli” OR “mountain goat” OR “Oreamnos americanus” OR “muskox*” OR “musk-ox*” OR “musk ox*” OR “Ovibos moschatus” OR “caribou” OR “Rangifer tarandus” OR “collared peccar*” OR “javelina*” OR “musk hog*” OR “musk-hog*” OR “Pecari tajacu” OR “white-lipped peccar*” OR “Tayassu pecari” OR “brocket*” OR “brown brocket*” OR “Mazama gouazoupira OR “red brocket*” OR “Mazama americana”) AND ("climat*" OR "global warming" OR “weather” OR "temperature" OR "precipitation" OR "snow*" OR “rain*” OR “ice” OR “icing” OR “drought” OR “heat” OR “cold” OR “freez*” OR “winter severity” OR “phenology”)).

The final Boolean search string was structured to capture articles that pertain to the population variables and exposure to direct climate variables (and their derivatives) or to climate-related changes in secondary variables.

We aimed to be inclusive with this search string and incorporated terms such as “climat*”, “global warming”, and “weather”, as well as terms for specific types of extreme climate events known to affect ungulates, such as drought and icing events. Based on the results of a scoping exercise detailed in our protocol [40], we deemed it unnecessary to include terms for the majority of our secondary climate variables. Because we were interested in all ungulate outcomes, we did not need to include outcome variables as terms. The comprehensiveness of our search string was assessed using a test list of articles known to be relevant to the authors (Additional file 2). The overall performance against the test list was 100% for Web of Science and 93% for Scopus.

#### Publication databases

We conducted our search using the online databases Web of Science and Scopus. In Web of Science, the Science Citation Index Expanded (SCI-EXPANDED), part of the Web of Science Core Collection, was searched. SCI-EXPANDED (1985–present) is the Core Collection citation index available to the authors via the U.S. Geological Survey. The search was run based on the “topic” field, which includes article titles, abstracts, keywords, and “KeyWords Plus” (automatically generated terms pulled from the titles of cited articles). In Scopus, all years of data were searched (1788–present), and searches were run on article titles, abstracts, and keywords. In both database searches, “present” was defined as September 2020.

#### Grey literature

We retrieved relevant grey literature through a combination of the Scopus search (using our search string) and manual hand-searches of relevant organization websites. Specifically, we searched the websites of each state, provincial, and territorial wildlife management agency in the U.S. and Canada (Additional file 3) to locate available technical reports on our focal ungulate species. Because our searches were in English only, we did not search the websites of Mexico’s or Greenland’s wildlife management agencies. A combination of methods was used to find relevant reports on websites. First, we hand-searched agency websites for pages containing reports and publications on ungulate species that are currently or have historically been in the state or province. We also entered search terms for those species (using species common names) into the general search function within each website, and in any search functions specific to reports and publications. For example, the Indiana Department of Natural Resources website was searched for “white-tailed deer”, the only ungulate species found in the state.

We compared reports that matched our review criteria to our list of articles retrieved during the database searches and downloaded any new papers. We categorized downloaded articles based on whether they (1) contained primary data relevant to our study criteria, (2) contained primary data relevant to our study criteria and cited potentially relevant sources, or (3) did not contain relevant primary data but cited potentially relevant sources. For articles that fell in categories 2 and 3, all potentially relevant citations were screened.

#### Supplementary searches

We searched the bibliographies of relevant review articles acquired during our searches of bibliographic databases and the websites of relevant organizations to ensure that any appropriate citations were captured and subjected to full text review. Review papers were only included in the map if they contained new primary data. The bibliographies of other articles that passed the screening process were not examined for relevant citations, due to time restrictions and the low likelihood of finding additional relevant studies not already captured. Lastly, we screened opportunistically retrieved articles that we encountered outside of our searches, such as those sent to us by colleagues.

#### Search results

The Web of Science and Scopus search results were de-duplicated in R v. 4.0.2 [47]. The de-duplicated results were then imported into the open access web-based platform Colandr, a machine learning tool that allows for iterative sorting of relevant and irrelevant articles [48], where they underwent title and abstract review. Articles identified during organization website searches were immediately screened and therefore bypassed the formal title and abstract and full-text review processes. Articles included during the snowballing of review paper bibliographies also bypassed title and abstract review and were only subjected to full-text review (see Screening process section). All accepted articles were stored in the open-source reference manager Mendeley (Mendeley Ltd.).

### Article screening and study eligibility criteria

#### Screening process

We carried out a two-step review process to assess the relevance of each article returned by our publication database searches: title and abstract review and full-text review. At the onset of the title and abstract review stage, six team members assessed the eligibility of a random subset of 100 articles and the level of agreement, or interrater reliability, was tested using the Fleiss Kappa statistic [49]. The kappa value was 0.75, indicating a substantial level of agreement between reviewers [50]. Following the completion of this exercise, the titles and abstracts of all articles were double screened by two reviewers in Colandr. Any questions about whether an article met screening criteria were discussed among reviewers. Bibliographic information of accepted articles from our database searches were exported into Microsoft Excel to create a list for full text review.

Articles from academic databases were assessed based on eligibility criteria and were single screened during full-text review, with the first 10% double screened to ensure consistency in application of the eligibility criteria. We recorded all articles excluded during full-text review, with reasons for exclusion provided (Additional file 4). We also maintained records of articles from our database searches that could not be acquired in a digital format via institutional journal subscriptions or interlibrary loan (Additional file 5). Eligible review articles that did not contain primary data were excluded from the systematic map. However, the bibliographies of these articles were screened, and articles not previously identified by the database searches but that met eligibility criteria were included in our map. Pre-screened eligible articles from organization websites were also added to our map. With the addition of grey literature, we made efforts to prevent the acquisition of data duplicates. For example, theses and dissertations were checked against accepted peer-reviewed articles. In cases of duplication, the peer-reviewed article was retained, and the thesis or dissertation was excluded. Articles published as corrections or comments to previously published articles were not included as new entries in our database. Rather, these follow-up articles were reviewed in conjunction with the original article and together were treated as a single combined entry. Reviewers who had authored an article under consideration were recused from decisions regarding the eligibility of the article.

#### Eligibility criteria

The eligibility of each article was assessed based on the criteria outlined below.

- *Eligible populations:* We included all subspecies and populations of wild pronghorn, elk, mule deer, moose, bighorn sheep (*Ovis canadensis*), white-tailed deer, American bison (*Bison bison*), mountain goat, Dall sheep (*Ovis dalli*), muskox, caribou, collared peccaries (*Pecari tajacu*), white-lipped peccaries (*Tayassu pecari*), brown brocket deer (*Mazama gouazoupira*), and red brocket deer (*M. americana*) in the U.S., Canada, Mexico, or Greenland. The state of Hawai’i, the U.S. territories of Puerto Rico, the U.S. Virgin Islands, Guam, the Northern Mariana Islands, and American Samoa, and the Canadian province of Prince Edward Island were excluded from the review. The 15 ungulate species are either not present on these islands or were introduced and are non-native.
- *Eligible exposures:* We included studies examining temporal changes in direct climate variables (i.e., temperature, precipitation, climate indices) and their derivatives (e.g., snow depth, winter severity, drought). Changes in direct climate variables can also affect ungulates via changes in what we termed “secondary variables”, such as plant phenology and disease transmission. For these secondary variables, we only accepted articles that attributed changes in a secondary variable (e.g., disease transmission) to changes in a direct climate variable (e.g., temperature), and examined the subsequent effects on ungulates (e.g., mortality).
- *Eligible comparators*: We included studies that made a comparison of at least two different time points, over which there was a quantified, inferred, or projected change in an exposure variable.
- *Eligible outcomes*: We included any and all effects on ungulates, such as physiology and condition, habitat and forage, population demographics, disturbances, migratory behavior, and seasonal range and corridor habitat.
- *Eligible study designs*: We included articles that explored quantified, inferred, or projected relationships between our exposure and outcome variables. We included articles that used observational, experimental, simulated, projected, or traditional ecological knowledge data.

### Study validity assessment

We did not carry out a formal study validity assessment as part of this effort. However, we coded information regarding the study design for each article (e.g., whether the relationship between the exposure variable(s) and outcome variable(s) was quantified, inferred, or projected), and users can consider this information when interpreting the studies in the map.

### Data coding and extraction strategy

Following full-text review, we extracted and entered information on each relevant article and the studies that comprised it into a spreadsheet database (Additional file 6). Here, we use the term “article” when describing a paper in its entirety and the term “study” when describing a research component of an article, noting that articles may be comprised of more than one study. For example, if an article examined climate effects on elk survival and mule deer survival in Yellowstone National Park, we considered this as two unique research components or “studies”, as this information was entered into unique rows in our database.

We extracted data from each study into the following high-level categories: (1) bibliographic information, (2) basic information about the study (i.e., study objective, study design, start and end dates, duration, and a qualitative determination of the extent to which climate was a primary focus of the study); (3) geographic information; (4) sections detailing the population, exposure (at two hierarchical levels), comparator, and outcome (at two hierarchical levels) variables; and (5) additional comments. We developed a standard format for data entry for each column, with codes used for some columns and open-ended text for others. Our lists of outcome and exposure variable codes were developed iteratively, and early on in the coding process we periodically expanded our options as we encountered new variables during data extraction. Previously coded papers were checked to determine whether coding should be modified to include any of the newly added codes.

Many of our exposure variables were coded using a “build a variable system”, whereby the reviewer engaged in a three-step process of coding a variable. First, the reviewer selected a capital letter code for the relevant climate variable (e.g., temperature = A), then selected a lower-case letter code describing how the variable was quantified (e.g., mean = x), and lastly selected a numeric code denoting the time scale at which the variable was measured (e.g., monthly = 3). In the above example, the resulting exposure variable entered in the database would be “Ax3”, to represent “mean monthly temperature”. This method proved to be an efficient approach for dealing with the dozens of exposure variables that were encountered during data extraction. For all fields, when information was missing or unclear, it was coded as not specified (“NS”) and when a field was not applicable for a study it was coded as “NA”. A detailed codebook (Additional file 7) was developed defining each database field, the appropriate format for data entry for each field, and the list of all codes and their corresponding definitions. Meanwhile, a two-tiered, nested structure was used to capture outcome variables. At the highest level (outcome level 1), all outcome variables were categorized as “physiology and condition”, “habitat and forage”, “population demographics”, “disturbances”, “migratory behavior”, or “seasonal range and corridor habitat”. They were then assigned a level-2 outcome (e.g., “reproductive success”, “body mass”), with each level-2 outcome being nested within the relevant level-1 outcome category.

Our database follows a relational structure, and therefore multiple rows were often used to enter data for a single article. For example, if an article examined different outcomes for male ungulates as compared to female ungulates, the information specific to males was entered in one row and the information specific to females was entered in second row. Other reasons for splitting data into multiple rows included articles with multiple study sites, multiple time periods, multiple species, differing seasonal information for exposure variables (e.g., spring precipitation and winter temperature), or differing seasonal information for outcome variables (e.g., winter mortality and annual survival). Data were distributed across two sheets in the database, with article information linked between sheets by a common numerical study ID. Data Sheet 1 contains bibliographic and study design information, while Data Sheet 2 contains all remaining article information. When an article needed to be split further in Data Sheet 1 (e.g., multiple study time periods), a unique letter was added to the numerical ID.

To ensure that data were extracted consistently, all 6 designated reviewers completed data entry for the first 10 articles. After each of the first 10 articles were entered, the team met to compare records and discuss any discrepancies and whether database changes were needed to facilitate consistency in data entry. The database structure was updated after each iteration, and the newest version of the database was used when testing the subsequent article. This process was carried out until all 10 articles were reviewed. Following completion of this exercise, articles were single screened during full-text review, with 10% double screened to ensure consistency in data entry. Any inconsistencies among reviewers were discussed until final decisions regarding data entry were agreed upon.

### Data mapping methods

Our primary product is a searchable, relational systematic map database, provided as a Microsoft Excel file (Additional file 6). All database columns can be filtered to return data that meet the user’s needs. The user can apply multiple database filters, for example filtering by species and outcome. Most users will likely be interested in filtering the database by species and or state/province. Field definitions are provided within the database via pop-up boxes on each column header.

Results are presented below as a series of statistics, bar charts, and heat maps to describe the abundance and distribution of evidence. We describe the quantity of evidence for particular species, exposures, and outcomes, highlighting evidence clusters and gaps pertaining to our primary research question.

## Review findings

### Review descriptive statistics

**Fig. 1:**
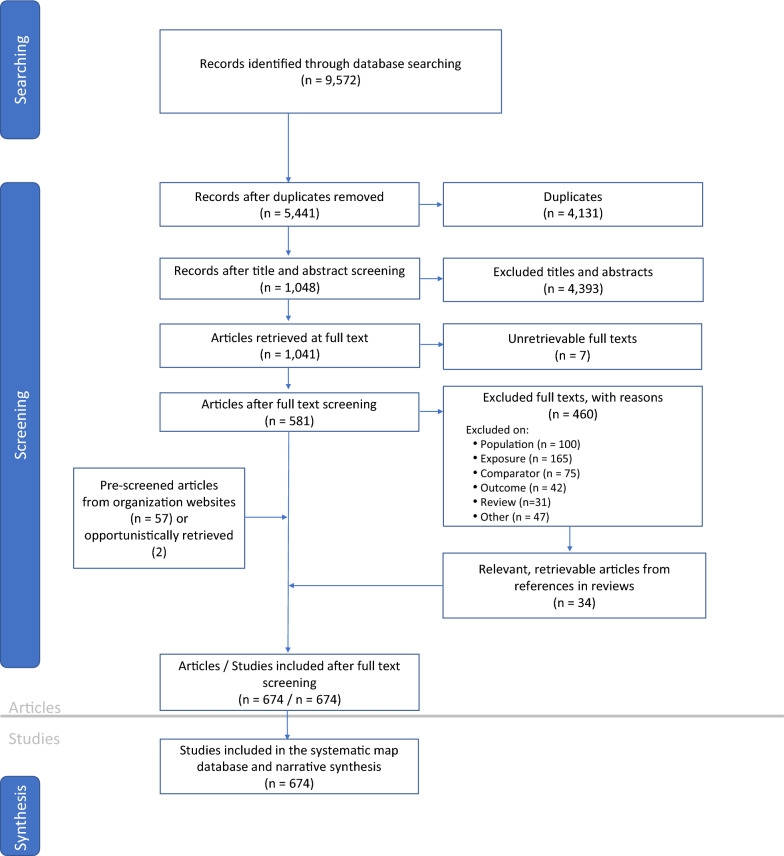
ROSES flow chart for the systematic map showing the number of records included at each stage of the review process

In total, 9572 articles were downloaded from the academic databases (Web of Science = 4522; Scopus = 5050) (Fig. 1). After duplicates were removed, 5441 articles remained and underwent our two-phase screening process. Of these, 4393 were excluded during the title and abstract review phase. An additional 460 articles were excluded during the full-text review phase and 7 articles were unretrievable at the full-text level. The first violation of PECO was selected as the reason for exclusion during full-text review. Reasons for exclusion are as follows: no eligible population (n = 100), no eligible exposure variable (n = 165), no eligible comparator (n = 75), no eligible outcome measured (n = 42), other miscellaneous reasons for exclusion, such as an ineligible language (n = 47), or, lastly, the article was a review paper that did not contain any primary research (n = 31). The latter were rejected and withheld to identify relevant articles cited in bibliographies.

Pre-screening of grey literature on organization websites identified in Additional file 3 yielded 57 eligible articles. Screening of the bibliographies of review papers resulted in the identification of 34 retrievable, relevant articles that had not been captured by our database or organization website searches. Lastly, we included two opportunistically retrieved articles. Ultimately, 674 articles were included in our systematic map (Additional file 6).

#### Article types

Of the 674 articles included in the final systematic map, 607 (90%) were academic papers and 67 (10%) were grey literature reports. Articles were published by 130 journals, 19 government agencies, and 7 dissertation and theses repositories. *The Journal of Wildlife Management* was the most frequently encountered publication source, with 115 of our included articles published by the journal.

#### Study design

**Fig. 2:**
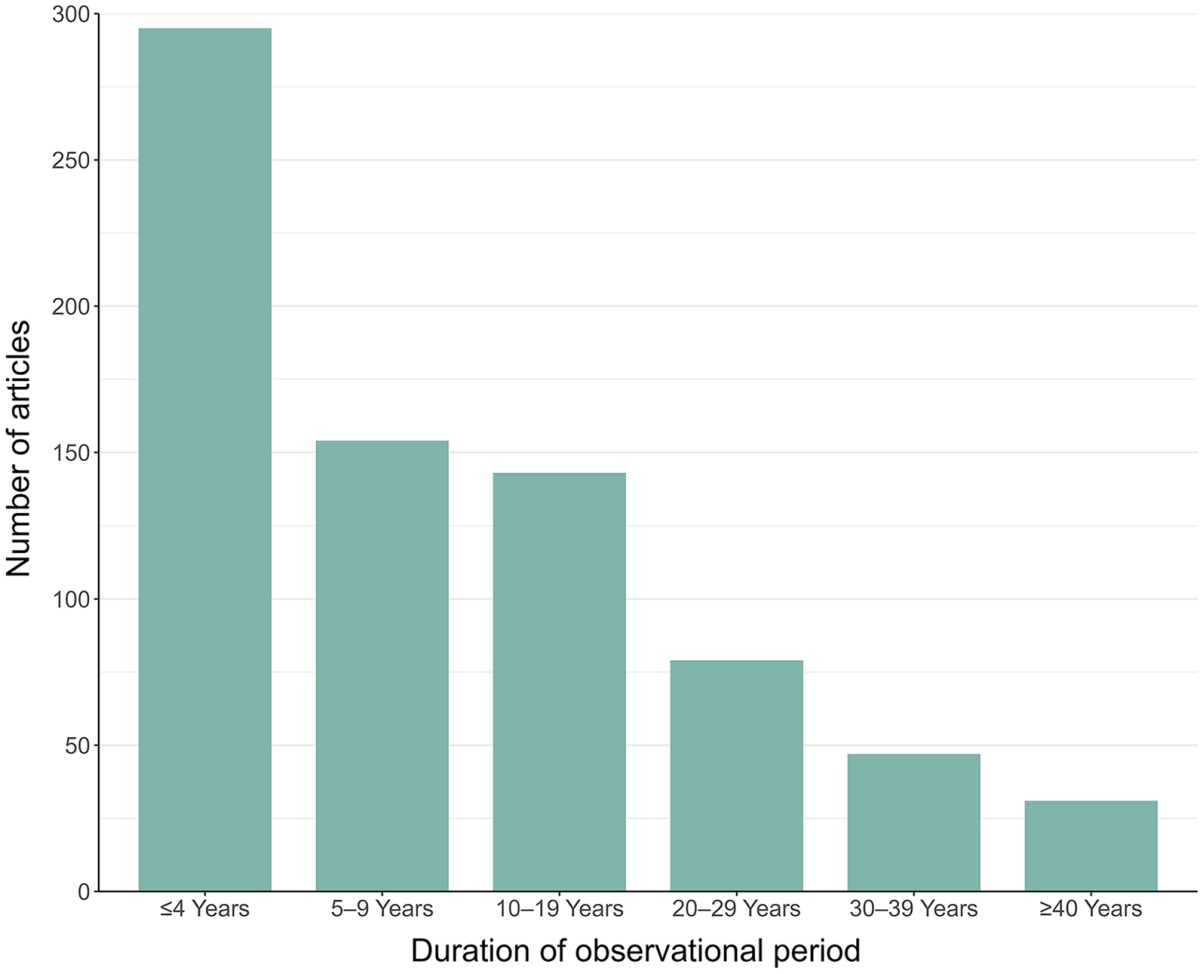
Duration of observational periods in articles

Noting that articles could have multiple study designs, the vast majority (95%) were classified as having an observational component. Some articles had multiple observational periods (e.g., if one population was monitored from 1994–1996 and a second population was monitored from 1995–1996). Observational periods were classified into duration categories, and individual studies were only represented once per category. Of the articles with observational studies, 295 had at least 1 observational period of 4 years in duration or shorter; 154 had at least 1 observational period of 5–9 years, and 143 had an observational period of 10–19 years. Notably, 31 articles had an observational period of ≥ 40 years (Fig. 2). This information is relevant for determining the proportion of articles that were examining short-term versus long-term changes in climate. Of the articles with projected studies (7%), 34% projected conditions for a period prior to 2050, and 52% projected conditions for 2051 or later. Some projected studies did not specify years. Additional study designs described in articles included those based on simulated data (4%), experimental data (3%), and Traditional Ecological Knowledge (0.3%). Some articles relied on multiple types of data, such as those that based projections on observational data.

Most studies directly quantified the relationship between climate variables and outcome variables (76%), although some studies inferred the relationship (24%) or projected the relationship based on future climate conditions (7%). This trend persisted when examining comparator types within each climate variable (i.e., temperature, precipitation, climate indices). Some studies included multiple methods for describing the relationship between exposure and outcome variables, such as those that quantified a relationship using observation data and projected a future relationship based on climate projections.

#### Temporal spread

**Fig. 3:**
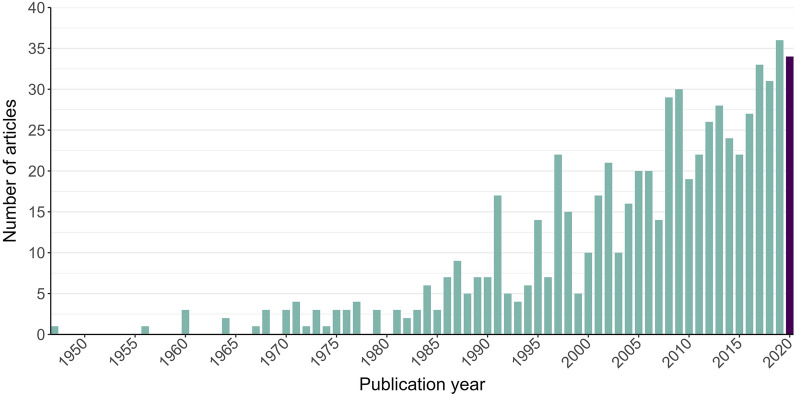
Number of articles published per year. Data for 2020 are incomplete, as searches were run in September 2020. One opportunistically retrieved article was published in 2021, and one was published in 2022; these articles were excluded from this figure as they were published outside of our formal search period

The number of relevant articles increased with each subsequent decade, from 1947 until September 2020, with the bulk of the evidence (88%) published in 1990 or later. There was considerable variation between years, particularly prior to 2010 (Fig. 3). For example, 20 articles were published in 2006, 14 articles in 2007, and 29 articles in 2008. The year with the greatest number of articles was 2019 (n = 36). The data for 2020 are incomplete, as our searches were run in late August and early September 2020. However, the number of articles published during the first 8–9 months of the year was 34, nearing the number of articles published during all of 2019.

#### Geographic spread

At the country level, the majority of articles were located in the U.S. (73%), followed by Canada (27%), Greenland (2%), and Mexico (2%), noting that some articles had study sites in multiple countries. Because our systematic map only included English-language articles, the geographic distribution of articles in our map are biased towards the U.S. and Canada (with the exception of Quebec). The number of articles generally decreased with increasing spatial scale. Most articles (76%) focused on a single study site, while 14% examined multiple study sites within a single state or province and 9% examined multiple study sites that spanned several states and provinces. Just over 1% of studies described their spatial extent as covering the focal species’ range, either within a single country or multiple countries.

**Fig. 4:**
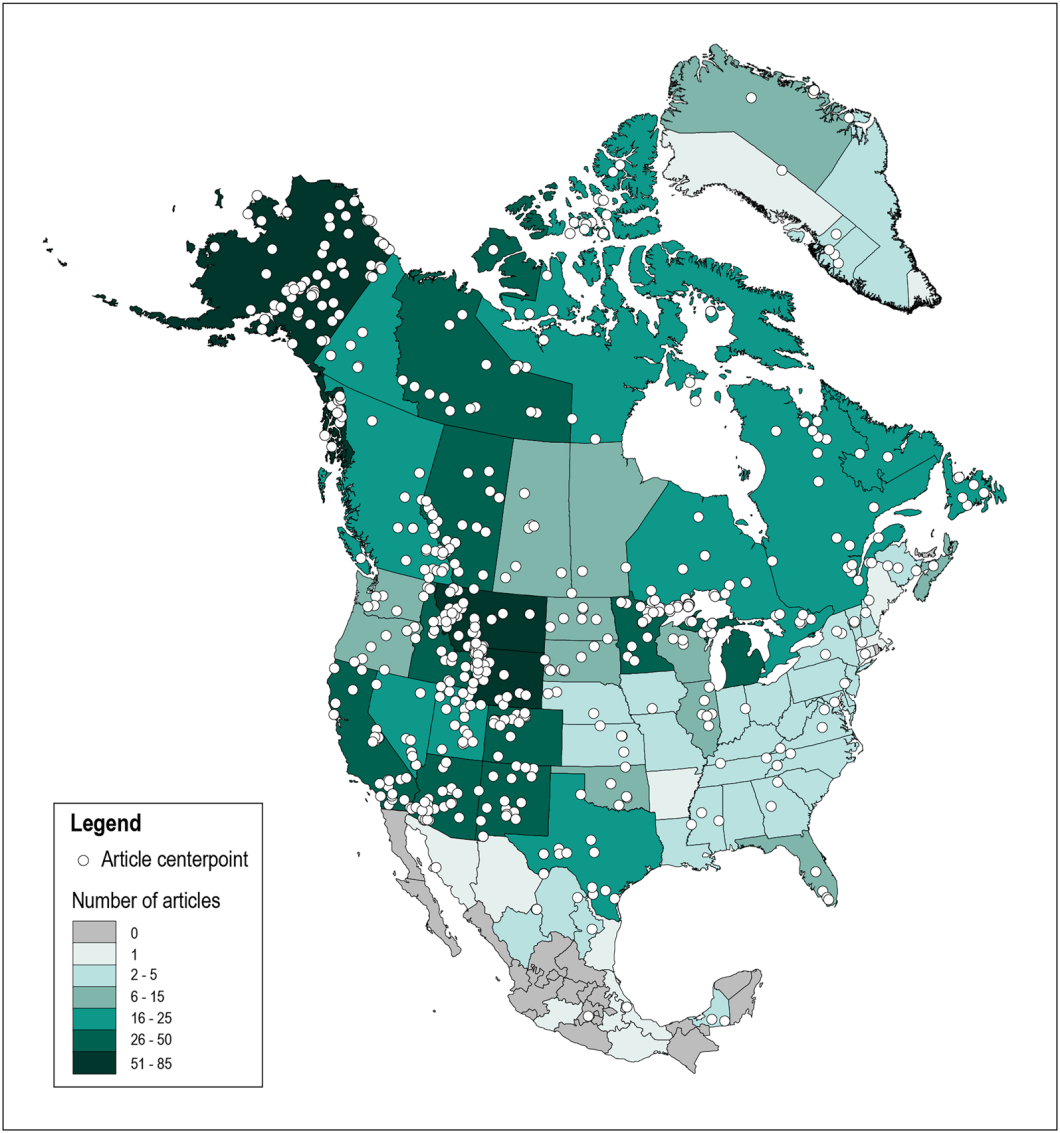
Map showing the geographic distribution of articles. Number of articles per state/province acquired based on location information provided in articles, rather than study centerpoints. Darker colors reflect more articles. Points represent article centerpoints. Some articles had multiple study sites from which centerpoints were calculated

At the state and province level, the locations with the greatest number of articles were the U.S. states of Wyoming (n = 85), Alaska (n = 82), and Montana (n = 74), followed by the Canadian province of Alberta (n = 45) (Fig. 4). Figure 4 shows the number of articles per state and province, with points representing the centerpoint location of each study. For articles that had multiple study sites, the mean center was extracted. Therefore, some of the points on the map do not represent the actual location of a study, but rather represent the centerpoint between two or more study sites. In our database, we detail how each article centerpoint was acquired (e.g., coordinates provided by the article, georeferenced from a figure in the article, or calculated from multiple study site coordinates). Figure 4 shows several identifiable hotspots of research located in the U.S. and Canadian Rocky Mountains; Interior, Southcentral, and Southeast Alaska; northern Minnesota and Michigan’s Upper Peninsula; and portions of the U.S. Southwest, including Southern California and southwestern Arizona.

### Mapping the quantity of studies relevant to the question

This systematic map primarily sought to understand what evidence exists on the effects of climate variability and climate change on ungulates in North America. This section outlines the species, exposure variables, and outcome variables that have been evaluated in the literature. For the majority of categories in our database, studies could be coded with multiple variables (e.g., both “temperature” and “precipitation”). Therefore, oftentimes the sum of percentages will be greater than 100 and the number of studies will not total 674.

#### Distribution of evidence by species

**Fig. 5:**
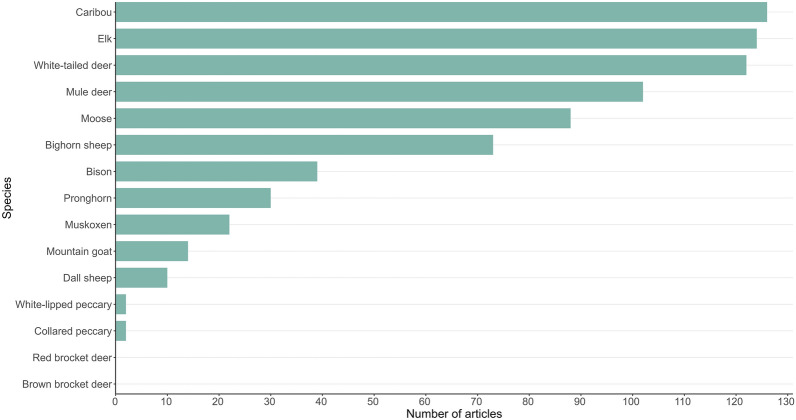
Number of articles per species

A total of 15 ungulate species are native to North America. Figure 5 shows the number of articles per species in our systematic map. Most articles (91%) focused on a single species, and the three most frequently studied species were caribou (n = 126), elk (n = 124), and white-tailed deer (n = 122). These were followed by mule deer (n = 102), moose (n = 88), and bighorn sheep (n = 73). There were no articles on brown brocket deer or red brocket deer and only two relevant articles were retrieved each for collared peccary and white-lipped peccary.

**Fig. 6:**
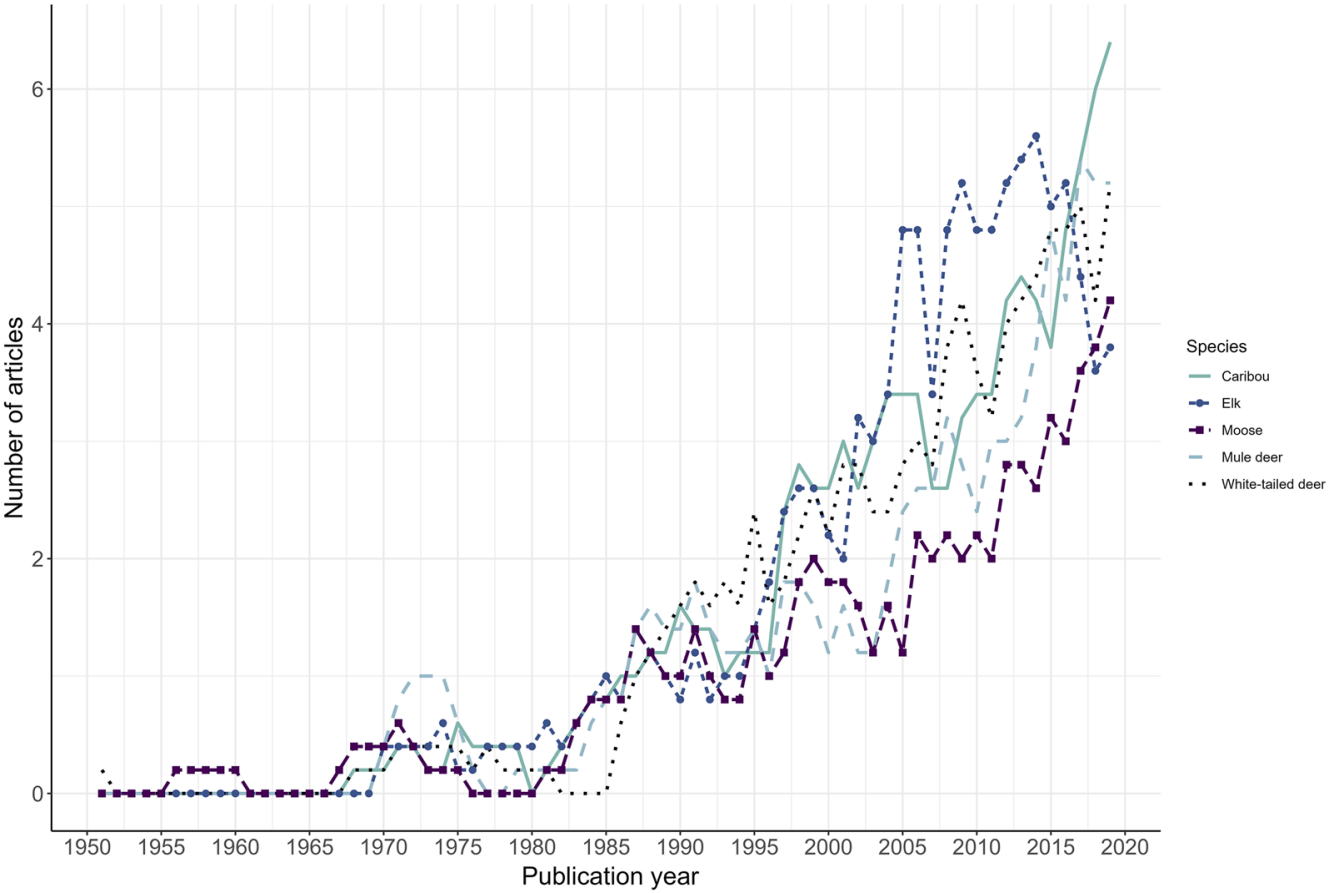
Temporal trends in the publication of literature for caribou, elk, moose, mule deer, and white-tailed deer from 1947–2019. Number of publications depicted based on a 5-year moving average. Data for 2020 are incomplete and as such were excluded from the average calculations

We also examined the temporal trends in the literature for the most frequently studied species. Figure 6 displays trends in the number of articles published per year for caribou, elk, mule deer, moose, and white-tailed deer based on a 5-year moving average. For all species, there is an overall increasing trajectory in the number of relevant articles published per year.

Lastly, we recorded the ungulate age and sex class examined in each article. Of the articles that specified sex class for one or more outcomes, the majority examined both females and males (n = 226) or females only (n = 220). Just 17 studies examined male ungulates only. Of the articles that specified age class for one or more study outcomes, most articles (n = 266) examined both adults and juveniles, 166 examined adults only, and 41 examined juveniles only.

#### Distribution of evidence by exposure variable

**Fig. 7:**
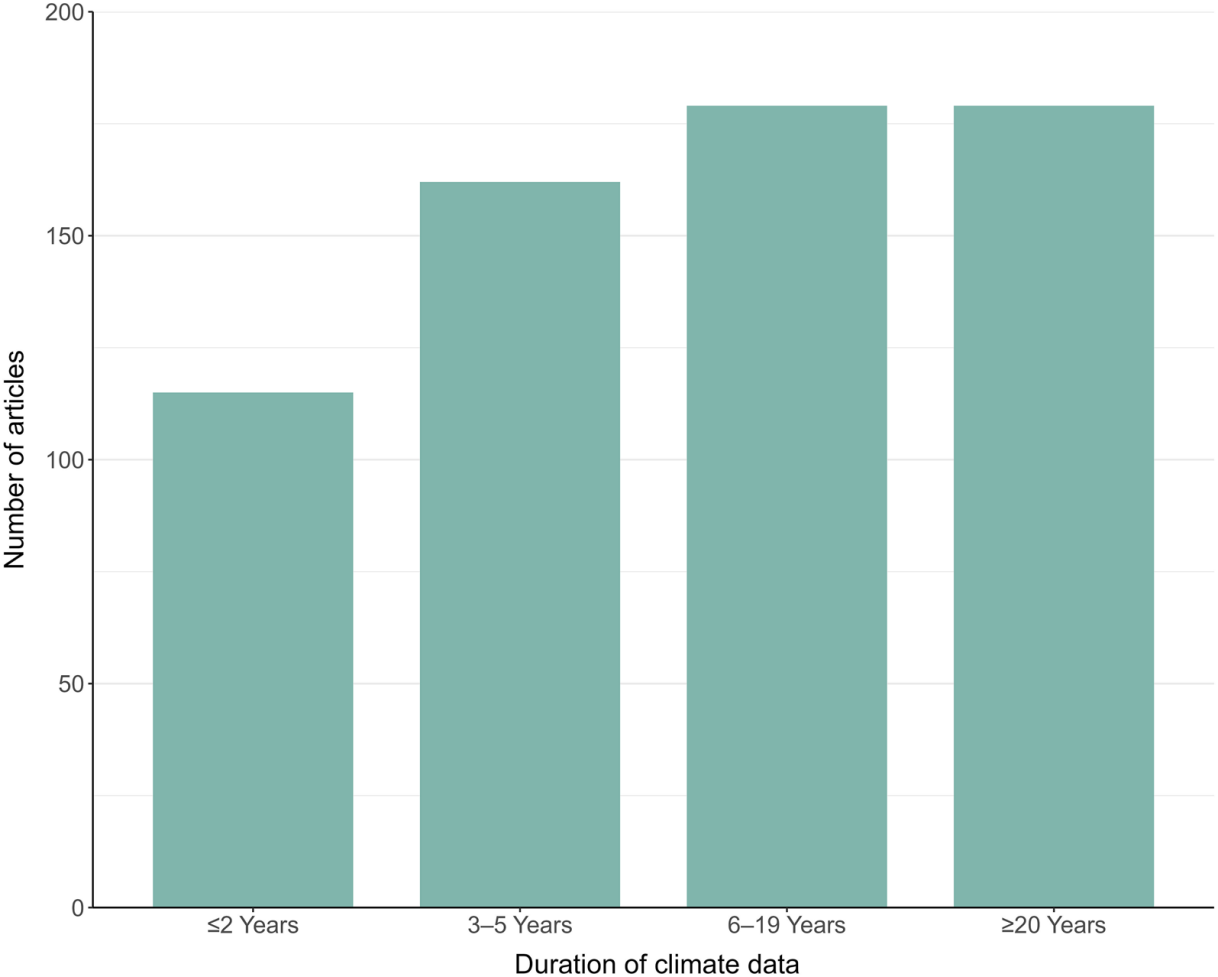
Duration of climate data used in articles with an observational component

Understanding the effects of climate on ungulate species was the primary focus of 76% of the articles included in our map, and a secondary focus in the remaining 24%. Articles assessing the long-term effects of climate change (≥ 20 years) were common, as were those assessing the shorter-term effects of climate variability (Fig. 7).

**Fig. 8:**
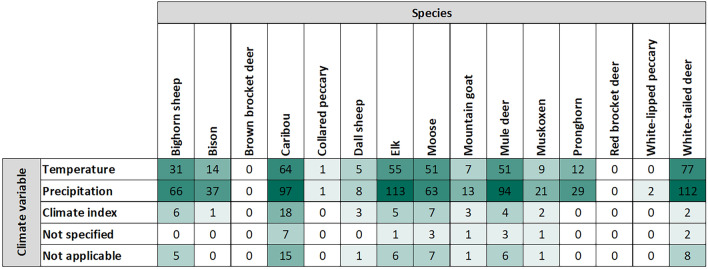
Number of articles per direct climate variable, by species

We examined exposure variables within a two-tier hierarchical structure. Each study was assigned at least one of three relevant “direct climate variables” (i.e., temperature, precipitation, climate index) and a “direct climate variable derivative", when provided (e.g., mean monthly temperature, snowpack, winter severity index). When examining results by direct climate variables, most articles (n = 588, 87%) looked at the effects of precipitation on ungulates, followed by temperature (n = 342, 51%). Other articles (n = 43, 6%) looked at the effects of large-scale climate indices such as the North Atlantic Oscillation Index and the Pacific Decadal Oscillation Index. When breaking down these results by species, precipitation was the most frequently examined climate variable for all species in our map, even those found in the rapidly warming Arctic, such as caribou and muskoxen (Fig. 8).

When exploring the more granular direct climate variable derivatives, the most frequently examined variables were ambient temperature, precipitation (type not specified), snow depth, and winter severity (Additional file 8). When combining all of the different snow related variables that we encountered (e.g., snow depth, snowpack, snow hardness), this becomes our most frequently represented type of climate variable (n = 354). Lastly, some studies looked at the effects of climate on ungulates indirectly through effects on secondary variables, most commonly predation, plant phenology (green-up), forage quantity, and forage quality.

#### Distribution of evidence by outcome variable

**Fig. 9:**
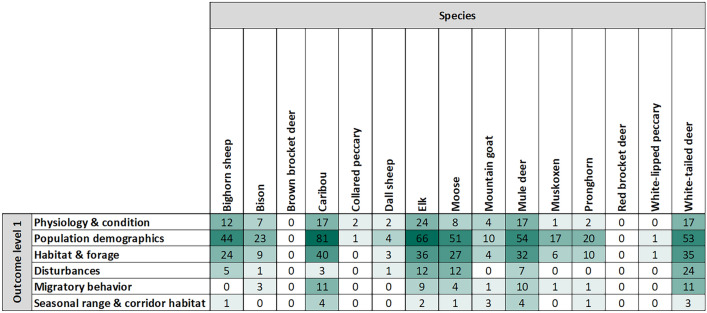
Number of articles per level-1 outcome variable, per species

We used a two-tier structure to categorize outcome variables, with 6 level-1 outcome variables and 67 level-2 outcome variables categorized within level-1 outcomes (Additional file 9). Of our level-1 outcomes, we most frequently encountered population demographic variables (n = 383, 57%), followed by habitat and forage variables (n = 202, 30%), and physiology and condition variables (n = 101, 15%). Disturbances, migratory behavior, and seasonal range and corridor habitat were the least studied level-1 outcome categories at 9%, 8%, and 3% of articles, respectively. For all species except collard peccary, population demographics was the most frequently examined level-1 outcome category, followed by the habitat and forage outcome category (Fig. 9). For each outcome category except seasonal range and corridor habitat, the relationship between exposure variables and outcome variables was most frequently quantified, followed by inferred, then projected. For example, the relationship between climate and population demographic outcomes was quantified in 286 instances, inferred in 88, and projected in 36.

Researchers evaluated outcomes within various seasons (i.e., annual, winter, spring, summer, fall). We determined the proportion of articles that evaluated combinations of outcomes and seasons out of the total number of articles that examined the corresponding outcome. Unique combinations were counted only once per article. The population demographic, physiology and condition, and disturbance variables were most frequently measured annually (50%, 36%, and 36%, respectively) and articles examining habitat and forage variables most frequently focused on winter conditions (51%). Migratory behavior variables were most frequently measured for the fall season (58%), followed by spring (39%), and the vast majority of seasonal range and corridor habitat research focused on winter range conditions (94%). Furthermore, for each level-1 outcome, more articles examined between-year changes than within-year changes in outcome variables.

Our level-2 outcome variables represent the most detailed outcome data that we recorded. When broken down by season, this list included 469 unique level-2 outcomes (Additional file 10). Of these, winter foraging- and habitat-related behavioral patterns (e.g., habitat selection, forage selection, home range size) was the most frequently encountered level-2 outcome (n = 82 occurrences). Within the physiology and condition category, the most frequently encountered variables were annual body mass/weight (n = 17) and winter nutritional condition (n = 15), while winter predation (n = 10) was the most frequently encountered disturbance outcome. In total, 63 articles (9%) examined a migration related variable. Departure timing in the fall (n = 14) and spring (n = 13) were the most frequently studied migratory behavior variables, and winter range location (n = 9) was the most frequently encountered seasonal range and corridor habitat variable. Within the population demographics category, the most frequently encountered variables were annual population productivity (n = 47), annual population growth rate (n = 37), annual adult survival (n = 37), and annual juvenile survival (n = 33). A total of 139 articles (21%) examined at least one survival or mortality variable. We also encountered 11 unique reproduction-related outcome variables and found that 63 articles (9%) examined at least 1 reproduction outcome.

We also examined how frequently the effects of each direct climate variable were assessed for each level-1 outcome variable (Additional file 11). Ambient temperature was the most frequently assessed variable in relationship to five out of six level-1 outcomes: physiology and condition (n = 53), population demographics (n = 173), habitat and forage (n = 85), disturbances (n = 32), and migratory behavior (n = 28). Precipitation (type not specified) was the second most frequently assessed variable in relationship to the physiology and condition (n = 43), population demographics (n = 138), and disturbances (n = 19) outcome variables, while snow depth was the second most frequently assessed variable for the habitat and forage (n = 69) and migratory behavior (n = 21) outcome variables. For the seasonal range and corridor habitat outcome, ambient temperature (n = 9) and snow depth (n = 9) were the most frequently assessed climate variables.

Lastly, we examined the sex and age class breakdowns across our level-1 outcome categories. We determined the proportion of articles that evaluated combinations of outcomes and age or sex class out of the total number of articles that examined the corresponding outcome. Unique combinations were only counted once per article. The combined males and females class was the most frequently reported sex class for the outcomes physiology and condition (39%), disturbances (37%), and seasonal range and corridor habitat (41%). “Females only” was the most frequently reported sex class for the population demographics (37%) and migratory behavior categories (57%). For the habitat and forage outcome category, an equal proportion of outcomes were measured for females only and both males and females combined (28%). When examining studied age classes, “adults only” was the most frequently represented age class among the habitat and forage (38%), physiology and condition (53%), population demographics (52%), and migratory behavior outcome categories (47%). The combined adults and juveniles class was the most frequently represented age class for the disturbances (34%), and seasonal range and corridor habitat outcomes (24%).

### Mapping the quality of studies relevant to the question

Mapping the quality of studies was not a goal of our systematic map. However, we recorded whether studies quantified or inferred the relationship between the exposure and outcome variables, as well as the duration of the study period in years. We also indicated any situation in which information pertaining to our database fields was unclear or missing from a study, using the code “NS” for “not specified”.

### Limitations of the map

#### Limitations due to search strategy

The goal of this systematic map was to identify all relevant evidence on the effects of climate variability and climate change on native ungulates in North America. However, due to resource limitations, we conducted our search using an English language search string, and only articles available in English were accepted into our map. Therefore, we were unable to include articles with study areas in Canada that were published in French only or articles in Mexico that were published in Spanish only. This likely resulted in an overall bias towards the U.S. and English-speaking Canadian provinces. Our search of organization websites for grey literature was particularly limited by our language restriction, as we were unable to hand search the websites of Mexico’s or Greenland’s wildlife management agencies. In addition, reports from the Quebec Ministry of Forests, Wildlife and Parks were only available in French and therefore excluded.

Furthermore, we were limited to literature that was available electronically, which resulted in the exclusion of books and some technical reports, theses, and dissertations. Literature available only in hard copy were often published prior to the 1980s, and therefore our approach may introduce a bias towards more recently published literature. Thirdly, we did not systematically screen the bibliographies of all relevant articles. Due to the large number of relevant articles in our map, we did not have the capacity to complete this exercise. Doing so likely would have resulted in the addition of some relevant articles and may have been particularly useful in identifying additional grey literature.

#### Limitations due to screening

It is important to emphasize that although we included articles exploring the effects of variables such as plant phenology, disease and parasitism, predation, and wildfire on ungulates, we only did so when articles examined the effects of climate on these secondary variables. Only then did we examine the effects of changes in secondary variables on ungulates. Therefore, this map does not represent a comprehensive record of studies on the effects of these secondary variables on ungulates. Doing so would have expanded the scope of this study well beyond climate effects on ungulates. Similarly, while we have a level-1 outcome category focused on habitat and forage, this map does not encompass all studies examining the effects of climate on habitat and forage species used by ungulates. We included these studies when they were retrieved by our final search string but did not attempt to comprehensively collate habitat and forage studies, as this would have required significant expansion of our search terms.

#### Limitations due to coding strategy

When coding direct climate variable derivatives as either “temperature” or “precipitation”, studies that described examining precipitation variables were only coded as such, even though temperature influences whether precipitation falls as rain, snow, or some other form. Our goal was to be as true as possible to a study when coding its information, and therefore we only coded studies as “temperature” if the study explicitly described examining a temperature variable.

Additionally, although we determined whether climate was a focus of each article, this was a subjective decision made by the reviewer after examining the goals and methods of an article, and there is no clear defining feature that separates the two categories. Therefore, these data are most useful for providing a sense of the extent to which climate was a focus of the body of literature as a whole, rather than for assessing individual studies.

Lastly, due to the large number of articles included in our map, we were unable to inquire about missing article information with article authors. In cases where article information for a database category was missing, we used the code “NS”, representing “not specified”.

## Conclusions

This systematic map catalogues the available evidence on the effects of climate variability and climate change on ungulates in North America. Our map captures 674 relevant articles published between 1947 and September 2020, with the rate of articles published increasing substantially during recent decades. The literature covered 13 of North America’s 15 native ungulate species, with caribou, elk, and white-tailed deer being the most frequently studied species. Geographically, more research has been conducted in the western U.S. and western Canada, though a notable concentration of research is also located in the Great Lakes region. Nearly 75% more articles examined the effects of precipitation on ungulates compared to temperature, with variables related to snow being the most commonly measured exposure variables. Most articles examined the effects of climate on ungulate population demographics, habitat and forage, and physiology and condition, with far fewer examining the effects on disturbances, migratory behavior, and seasonal range and corridor habitat.

### Implication for policy/management

The effects of climate change, and its interactions with stressors such as land-use change, disease, predation, and invasive species, is of increasing concern to wildlife managers [13, 15]. This systematic map identifies numerous areas of research that can contribute to an improved understanding of how changing climate conditions have already and could potentially affect ungulates in North America. For the 13 ungulate species for which we acquired literature, researchers have explored how climate variability and climate change impact 67 outcomes, ranging from antler size to population abundance to the timing of migration. Articles examining climate impacts on ungulate population demographics are particularly abundant, and there are more than 80 relevant articles each for caribou, elk, white-tailed deer, mule deer, and moose. Ungulate managers at state/province, tribal territory, and local scales can use this systematic map to quickly identify evidence relevant to their species and location of interest, with the ability to further refine selections based on specific climate impacts or outcome variables of interest.

Understanding the impacts of external stressors like climate change and climate variability on life-histories and population dynamics in particular is foundational to maintaining viable and sustainable ungulate populations in North America. Decisions regarding population control measures (e.g., harvest limits); supplemental feeding; translocation, introduction, and re-introduction efforts; and the application of habitat treatments represent just some of the management decisions that can be informed by an improved understanding of climate impacts. Our systematic map enables managers to identify relevant literature on climate impacts on ungulates in order to determine potential management strategies. For example, evidence has demonstrated that severe winter weather can negatively impact some pronghorn populations [51]. This information can help managers target habitat treatments in areas of pronghorn range where increased winter storm frequency is projected, to help sustain populations through winter.

Climate impacts on ungulates can also have implications for the broader landscape. For example, white-tailed deer in the Southeast U.S. were shown to be more selective of plant species during drought, which could impact the persistence of commonly eaten deer forage, even in cases where deer density does not exceed typical natural carrying capacity [52]. Understanding interactions between species and habitat under changing climate conditions can inform habitat treatment planning and population control measures.

Identifying the impacts of climate on the winter- and summer-range habitats of migratory populations is also of importance to managers. Changes to seasonal range conditions can affect the availability of quality forage, which in turn has implications for ungulate condition, reproductive success, and survival [53]. Climate variability and climate change can also affect the timing of ungulate migration and the decision to migrate, through its effects on forage and key weather events, with numerous management implications [30]. For example, white-tailed deer density can be estimated using aerial surveys of winter ranges depending on land cover and deer behavior. However, among conditional migrants, snow depth can determine whether deer choose to migrate to winter range [54]. Understanding this relationship can help managers select the most appropriate survey methods that consider the year’s winter conditions. Behavioral responses to climate change can also have cascading effects on ecosystems, such as when changes in ungulate distribution on seasonal habitats lead to altered browsing pressure and seed dispersal [55]. For example, in montane Arizona, where mountain snowfall has decreased over a 25-year period, Martin and Maron [56] experimentally demonstrated that the observed decrease in deciduous trees and associated songbirds was a result of increased winter browsing by elk, rather than the direct effects of snowfall on woody vegetation. Therefore, the effects of climate change on ungulates also have implications for broader effects on ecosystems.

With its expansive scope, this systematic map enables ungulate managers in North America to bypass the time-intensive step of searching for articles that are relevant to the populations they manage. Managers can use this map to efficiently identify literature documenting climate impacts on ungulate species and populations of interest and can review these findings to help prepare for future changes to the populations they manage, as climate conditions continue to change.

### Implication for research

#### Knowledge clusters

Our map identifies clusters of information that warrant deeper examination via systematic reviews. Because the exposure and response to climate variability and climate change can vary by population, syntheses that are species-specific or focused on particular geographic regions would likely be of most value to managers. Our map highlights that caribou, elk, and white-tailed deer have the highest number of articles (n ≥ 122 each), followed by mule deer, moose, and bighorn sheep (n ≥ 73 each). However, bison (n = 39), pronghorn (n = 30), and muskoxen (n = 22) also potentially have sufficient evidence available to synthesize in a review. Researchers could also examine climate impacts on one or more ungulate species found in regions with concentrations of evidence, such as the Rocky Mountains (U.S. and Canada), Alaska, the Great Lakes region, and the Southwest U.S. Alternatively, reviews focused on specific species in a specific region could also be carried out, such as elk and mule deer in the Greater Yellowstone Ecosystem. A systematic review focused on climate impacts on ungulates in the rapidly warming Arctic could also be valuable to prioritize.

The majority of our level-1 outcome categories have sufficient evidence to synthesize and would also be appropriate focal topics for future reviews. Population demographics (n = 384), habitat and forage (n = 202), and physiology and condition (n = 101) in particular have substantial evidence and could be further examined by species and location. Because ungulate population size and trend data can be used to inform ungulate management decisions, a review of climate effects on population demographics would be particularly valuable.

Although migratory behavior was one of our less well-studied outcome categories (n = 51), one clear trend among these articles is that the majority (77%) examined the impacts of snow on migration. Snow is an important driver of seasonal ungulate migrations in North America, with increasing snowfall and decreasing temperatures driving the timing of fall migration and decreasing snow depth and increasing plant growth being drivers of spring migration [29]. A systematic review of the effects of snow on fall and spring migration could provide insight into the relevance of this variable across species and geographies, and how the timing of migration might change in regions that are anticipated to experience changes in winter precipitation.

Lastly, our map identifies that there is a large base of observational studies that could be used to model projected impacts of climate change on ungulates. Forward-looking studies could help managers understand how and when long-term management strategies may need to be adjusted. For example, our map demonstrates that more articles evaluated the effects of precipitation as compared to temperature on ungulate outcomes, which may be beneficial given uncertainty associated with projecting changes in precipitation. Greater uncertainty in future precipitation conditions is thought to be a result of the lower importance of greenhouse gas emission scenarios for understanding precipitation than temperature. Variation in precipitation is more commonly a result of climate variability, which can lead to irreducible uncertainty in both the short- and long-term [57]. While there is confidence that certain regions of North America will become warmer or dryer (e.g., [58]), understanding how climate variability may manifest at local scales is an ongoing challenge [57]. Thus, an emphasis on observed precipitation variation in ungulate studies can provide insights on how a range of rain and snowfall scenarios may affect populations and assist managers with making more robust decisions regarding future environmental change.

#### Knowledge gaps

This map also identifies several understudied topics that would benefit from additional primary research. First, we found fewer articles exploring changes in ungulate migration than expected (9%), considering the documented links between weather and climate and migration. The lack of migration studies relative to other outcome categories is likely due to a combination of factors. First, data-driven ungulate management decisions are based on population size and trend, contributing to the observed emphasis on demographics studies over migration studies. Second, many ungulate populations are non-migratory, and while life-history and population dynamic variables are important to understand for all populations, migration variables are not consistently relevant across populations. Last, there was a lack of wide-spread availability of GPS technology for animal tracking prior to the 1990s. Subsequent advancements in the technology have enabled researchers to map migration with increasing precision in recent decades [29], and the increasing rate of migration studies since around the year 2000 may be due in part to the increased application of GPS technology. While our map demonstrates that the frequency of migration studies is continuing to increase, it is worth emphasizing the importance of continuing to explore climate impacts on migration. Factors such as the decision to migrate and the timing of migration are directly and indirectly tied to weather and climate, with winter severity being a key driver of fall migration and plant phenology being a driver of spring migration [29]. Understanding baseline migratory patterns, as well as changes to these patterns, will be important for managers as climate conditions continue to change.

We also identified disparities in the number of articles for each species. The frequency with which each species is studied is likely due to a range of factors, including population size and trend, conservation status, intensity of management, and sensitivity to climate change. In the context of climate sensitivity, we identified several species that are relatively understudied. We found only 30 articles on pronghorn, a species known to be particularly sensitive to even small changes in forage conditions and which has exhibited large population fluctuations due to drought and severe winters [59]. Additional articles focused on pronghorn across their range, which stretches from Mexico into the Rocky Mountain and Great Basin regions [60], would improve our understanding of the effects of climate change on this species. Similarly, we found only 14 articles on mountain goats. Cold adapted alpine species are particularly sensitive to variations in climate, such as warm summer temperatures [61], therefore additional mechanistic studies examining the effects of climate variation on mountain goat life-histories and population dynamics would be of value. Lastly, we found only two relevant articles for collared peccary and white-lipped peccary and no relevant articles on brown brocket or red brocket deer. This may be due in part to biases in our search strategy and the species’ comparatively limited North American range. The absence of climate studies on brocket deer in particular may also reflect the overall lack of available scientific information on tropical deer populations in Mexico [62]. Although more information exists on peccaries in Mexico, most of the studies we encountered examined the effects of wet and dry season conditions on peccaries, versus changes in climate over time, and were excluded for that reason. Identifying true geographic gaps in the literature is challenging, as numerous factors likely contribute to the quantity of research being carried out on this topic in each state or province. First, the number of ungulate species and their abundance varies across the continent. We would generally expect that states and provinces with fewer ungulate species would have less ungulate research overall. For example, 8 ungulate species are found in Wyoming, a state with 85 relevant articles; in contrast, 2 ungulate species are found in Arkansas, a state with 1 relevant article. The location of studies may also be impacted by the rate at which a particular region is experiencing changes in climate. We found concentrations of evidence in the Southwest U.S., a region which is experiencing intensifying droughts; in the Northern Great Plains, a region experiencing more frequent extreme precipitation events; and in Alaska, which is warming faster than any other state [63–65]. There is comparatively less research focused on the eastern U.S., with most states in the region having 1–5 relevant articles and being home to 1–2 ungulate species. White-tailed deer are the predominant ungulate species found in the region, and their overabundance has resulted in extensive damage to agriculture and native vegetation and the transmission of infectious agents [66]. Evidence suggests that climate change will favor white-tailed deer populations in some regions, such as the Midwest (e.g. [15]), and conducting additional research on how changes in climate could affect deer demographics throughout these regions will be important for understanding the future effects of this species on ecosystems, agriculture, and human and wildlife health. Lastly, our results appear to highlight a limited evidence base in Mexico. However, we are unable to estimate the extent to which this is a biproduct of our English-language search terms versus an expression of a true data gap.

Finally, we identified a gap in long-term studies assessing ungulate responses to climate change. Nearly half of all observation periods were less than 10 years in duration, with many being less than or equal to 4 years. These shorter-term observations can provide important insights into the potential effects of multidecadal deviations from average temperature and precipitation when longer term studies are unavailable. Short-term studies can be compared to similar short-term studies on the same population occurring years or decades apart, which can provide insight into the effects of longer-term changes in climate on a population. However, long-term studies are needed to fully understand and predict responses to climate change. For example, studies measuring species range shifts produce noisy estimates when they take place over the short-term, due to natural variability in range limits and time lags in species responses [67]. Moreover, longer-term studies are needed to understand whether short-term responses to changing climates are adaptive in the long-term [68]. For example, different phenological shifts between species in response to changing temperatures can alter interspecies interactions and competition dynamics [69].

Although gaps in the evidence remain, our systematic map demonstrates an encouraging increasing trend in the number of articles examining the effects of climate variability and climate change on ungulates in North America. Future research that seeks to fill existing knowledge gaps, alongside systematic reviews that target identified knowledge clusters, can inform proactive ungulate management strategies in an era of global change.

## Supplementary Information

**Additional file 1.** ROSES for systematic map reports.

**Additional file 2.** Results of the sensitivity testing of the final search string and the list of articles used to test the sensitivity of the search string.

**Additional file 3.** Management agency websites hand-searched for grey literature.

**Additional file 4.** Articles excluded during full-text review.

**Additional file 5.** Unobtained articles.

**Additional file 6.** Article database.

**Additional file 7.** Database codebook.

**Additional file 8.** Number of articles with at least one study per direct climate variable derivative and secondary climate variable.

**Additional file 9.** List of outcome variables used in the systematic map.

**Additional file 10.** Distribution of articles with at least one study among outcome variables, split by seasons for which outcomes were measured.

**Additional file 11.** Distribution of articles with at least one study among level-1 outcome and exposure variables.

## Data Availability

All data generated and analyzed during this study are included in this published article and its additional files. The article database is also available at: Malpeli, K.C., Endyke, S.C., Weiskopf, S.R., Thompson, L.M., Johnson, C.G., and Carlin, M.A., 2024, Catalogue of the literature assessing climate effects on ungulates in North America (1947–2020): U.S. Geological Survey data release, https://doi.org/10.5066/P1EMV7HO.
