# Supplementary material for: Existing evidence on the effects of climate variability and climate change on ungulates in North America: a systematic map
Source: Environ Evid. 2024 Apr 4;13:8. doi: 10.1186/s13750-024-00331-8 (PMC11378825; doi:10.1186/s13750-024-00331-8)
Supplement: Supplementary file 2 — Additional file 2. Results of the sensitivity testing of the final search string and the list of articles used to test the sensitivity of the search string. [file 13750_2024_331_MOESM2_ESM.docx]

**Additional file 2. Results of the sensitivity testing of the final search string and the list of articles used to test the sensitivity of the search string.**

Results of sensitivity testing

| ***Web of Science*** | | ***Scopus*** | |
| --- | --- | --- | --- |
| **Total number of articles returned** | **Percent of test list articles returned** | **Total number of articles returned** | **Percent of test list articles returned** |
| 4,522 | 100% (30/30) | 5,050 | 93% (28/30) |

Articles used for sensitivity testing

1. Aikens EO, Kauffman MJ, Merkle JA, Dwinnell SPH, Fralick GL, Monteith KL. The greenscape shapes surfing of resource waves in a large migratory herbivore. Ecol Lett. 2017;20:741–50. Available from: <https://doi.org/10.1111/ele.127722>.
2. Bender LC, Lomas LA, Browning J. Condition, Survival, and Cause-Specific Mortality of Adult Female Mule Deer in North-Central New Mexico. J Wildl Manage. 2007;71:1118–24. Available from: <https://doi.org/10.2193/2006-2263>.
3. Bowyer RT, Van Ballenberghe V, Kie JG. Timing and Synchrony of Parturition in Alaskan Moose: Long-Term Versus Proximal Effects of Climate. J Mammal. 1998;79:1332–44. Available from: <https://doi.org/10.2307/13830254>.
4. Bright JL, Hervert JJ. Adult and fawn mortality of Sonoran pronghorn. Wildl Soc Bull. 2005;33:43–50. Available from: [https://doi.org/10.2193/0091-7648(2005)33[43:AAFMOS]2.0.CO5](https://doi.org/10.2193/0091-7648(2005)33%5b43:AAFMOS%5d2.0.CO5).
5. Brown DE, Warnecke D, McKinney T. Effects of midsummer drought on mortality of doe pronghorn (Antilocapra Americana). Southwest Nat. 2006;51:220–5. Available from: [https://doi.org/10.1894/0038-4909(2006)51[220:EOMDOM]2.0.CO6](https://doi.org/10.1894/0038-4909(2006)51%5b220:EOMDOM%5d2.0.CO6).
6. Caltrider T, Bender LC. Relationships Between Landscape Greenness and Condition of Elk, Mule Deer, and Pronghorn in New Mexico. Rangel Ecol Manag. 2018;71:259–65. Available from: <http://www.sciencedirect.com/science/article/pii/S15507424173011007>.
7. Christie KS, Jensen WF, Schmidt JH, Boyce MS. Long-term changes in pronghorn abundance index linked to climate and oil development in North Dakota. Biol Conserv. 2015;192:445–53. Available from: <http://www.sciencedirect.com/science/article/pii/S00063207153015558>.
8. Craine JM, Towne EG, Joern A, Hamilton RG. Consequences of climate variability for the performance of bison in tallgrass prairie. Glob Chang Biol. John Wiley & Sons, Ltd; 2009;15:772–9. Available from: <https://doi.org/10.1111/j.1365-2486.2008.01769.x9>.
9. Dawe KL, Bayne EM, Boutin S. Influence of climate and human land use on the distribution of white-tailed deer (Odocoileus virginianus) in the western boreal forest. Can J Zool. NRC Research Press; 2014;92:353–63. Available from: https://doi.org/10.1139/cjz-2013-026210.
10. Eggeman SL, Hebblewhite M, Bohm H, Whittington J, Merrill EH. Behavioural flexibility in migratory behaviour in a long-lived large herbivore. J Anim Ecol. 2016;85:785–97. Available from: <https://doi.org/10.1111/1365-2656.1249511>.
11. Epps CW, McCullough DR, Wehausen J, Bleich VC, Rechel JL. Effects of Climate Change on Population Persistence of Desert-Dwelling Mountain Sheep in California. Conserv Biol. 2004;18:102–13. Available from: <https://doi.org/10.1111/j.1523-1739.2004.00023.x12>.
12. Gedir J V, Cain JW, Harris G, Turnbull TT. Effects of climate change on long-term population growth of pronghorn in an arid environment. Ecosphere. 2015;6:art189. Available from: <https://doi.org/10.1890/ES15-00266.113>.
13. Hebblewhite M. Predation by wolves interacts with the North Pacific Oscillation (NPO) on a western North American elk population. J Anim Ecol. 2005;74:226–33. Available from: <https://doi.org/10.1111/j.1365-2656.2004.00909.x14>.
14. Joly K, Duffy PA, Rupp TS. Simulating the effects of climate change on fire regimes in Arctic biomes: implications for caribou and moose habitat. Ecosphere. John Wiley & Sons, Ltd; 2012;3:art36. Available from: <https://doi.org/10.1890/ES12-00012.115>.
15. Kucera TE. Influences of sex and weather on migration of mule deer in California. Gt Basin Nat. 1992;52:122–30. Available from: <http://www.jstor.org/stable/4171270516>.
16. Long RA, Bowyer RT, Porter WP, Mathewson P, Monteith KL, Kie JG. Behavior and nutritional condition buffer a large-bodied endotherm against direct and indirect effects of climate. Ecol Monogr. 2014;84:513–32. Available from: <https://doi.org/10.1890/13-1273.117>.
17. Merkle JA, Monteith KL, Aikens EO, Hayes MM, Hersey KR, Middleton AD, et al. Large herbivores surf waves of green-up during spring. Proc R Soc B Biol Sci. 2016;283:20160456. Available from: <https://doi.org/10.1098/rspb.2016.045618>.
18. Middleton AD, Kauffman MJ, McWhirter DE, Cook JG, Cook RC, Nelson AA, et al. Animal migration amid shifting patterns of phenology and predation: lessons from a Yellowstone elk herd. Ecology. 2013;94:1245–56. Available from: <https://doi.org/10.1890/11-2298.119>.
19. Middleton AD, Merkle JA, McWhirter DE, Cook JG, Cook RC, White PJ, et al. Green-wave surfing increases fat gain in a migratory ungulate. Oikos. 2018;127:1060–8. Available from: <https://doi.org/10.1111/oik.0522720>.
20. Monteith KL, Bleich VC, Stephenson TR, Pierce BM, Conner MM, Kie JG, et al. Life-history characteristics of mule deer: Effects of nutrition in a variable environment. Wildl Monogr. 2014;186:1–62. Available from: <https://doi.org/10.1002/wmon.101121>.
21. Monteith KL, Bleich VC, Stephenson TR, Pierce BM, Conner MM, Klaver RW, et al. Timing of seasonal migration in mule deer: effects of climate, plant phenology, and life-history characteristics. Ecosphere. 2011;2:art47. Available from: <https://doi.org/10.1890/ES10-00096.122>.
22. Monteith KL, Klaver RW, Hersey KR, Holland AA, Thomas TP, Kauffman MJ. Effects of climate and plant phenology on recruitment of moose at the southern extent of their range. Oecologia. 2015;178:1137–48. Available from: <https://doi.org/10.1007/s00442-015-3296-423>.
23. Pettorelli N, Pelletier F, Hardenberg A von, Festa-Bianchet M, Côté SD. Early onset of vegetation growth vs. rapid green-up: Impacts on juvenile mountain ungulates. Ecology. 2007;88:381–90. Available from: <https://doi.org/10.1890/06-087524>.
24. Poole KG, Mowat G. Winter habitat relationships of deer and elk in the temperate interior mountains of British Columbia. Wildl Soc Bull. John Wiley & Sons, Ltd; 2005;33:1288–302. Available from: [https://doi.org/10.2193/0091-7648(2005)33[1288:WHRODA]2.0.CO25](https://doi.org/10.2193/0091-7648(2005)33%5b1288:WHRODA%5d2.0.CO25).
25. Rickbeil GJM, Merkle JA, Anderson G, Atwood MP, Beckmann JP, Cole EK, et al. Plasticity in elk migration timing is a response to changing environmental conditions. Glob Chang Biol. 2019;25:2368–81. Available from: <https://doi.org/10.1111/gcb.1462926>.
26. Simpson DC, Harveson LA, Brewer CE, Walser RE, Sides AR. Influence of Precipitation on Pronghorn Demography in Texas. J Wildl Manage. 2007;71:906–10. Available from: <https://doi.org/10.2193/2005-75327>.
27. Singer FJ, Harting A, Symonds KK, Coughenour MB. Density Dependence, Compensation, and Environmental Effects on Elk Calf Mortality in Yellowstone National Park. J Wildl Manage. 1997;61:12–25. Available from: <http://www.jstor.org/stable/380241028>.
28. Wang G, Thompson Hobbs N, Singer FJ, Ojima DS, Lubow BC. Impacts of Climate Changes on Elk Population Dynamics in Rocky Mountain National Park, Colorado, U.S.A. Clim Change. 2002;54:205–23. Available from: <https://doi.org/10.1023/A:101572510334829>.
29. White KS, Gregovich DP, Levi T. Projecting the future of an alpine ungulate under climate change scenarios. Glob Chang Biol. John Wiley & Sons, Ltd; 2018;24:1136–49. Available from: <https://doi.org/10.1111/gcb.1391930>.
30. White KS, Pendleton GW, Crowley D, Griese HJ, Hundertmark KJ, Mcdonough T, et al. Mountain goat survival in coastal Alaska: Effects of age, sex, and climate. J Wildl Manage. John Wiley & Sons, Ltd; 2011;75:1731–44. Available from: https://doi.org/10.1002/jwmg.238
