# Supplementary material for: Existing evidence on the effects of climate variability and climate change on ungulates in North America: a systematic map
Source: Environ Evid. 2024 Apr 4;13:8. doi: 10.1186/s13750-024-00331-8 (PMC11378825; doi:10.1186/s13750-024-00331-8)
Supplement: Supplementary file 3 — Additional file 3. Management agency websites hand-searched for grey literature. [file 13750_2024_331_MOESM3_ESM.docx]

**Additional file 3: Management agency websites hand-searched for grey literature.**

| **Management agency** | **URL** | **Date searched** |
| --- | --- | --- |
| ***Canada*** | | |
| Alberta Ministry of Environment and Parks | <https://www.alberta.ca/environment-and-parks.aspx> | 8/26/2020 |
| British Columbia Ministry of Forests, Lands and Natural Resource Operations, Fish and Wildlife Branch | <http://www.env.gov.bc.ca/fw/> | 8/27/2020 |
| Manitoba Fish and Wildlife | <https://www.gov.mb.ca/sd/fish_and_wildlife/wildlife/index.html> | 8/28/2020 |
| New Brunswick Department of Natural Resources | <https://www2.gnb.ca/content/gnb/en/departments/erd/natural_resources.html> | 8/28/2020 |
| Newfoundland and Labrador Department of Fisheries and Land Resources | <https://www.gov.nl.ca/flr/> | 8/28/2020 |
| Northwest Territories Department of Environment and Natural Resources | <https://www.enr.gov.nt.ca/en> | 8/28/2020 |
| Nova Scotia Department of Lands and Forestry | <https://novascotia.ca/natr/> | 8/31/2020 |
| Nunavut Department of Environment | <https://www.gov.nu.ca/environment> | 8/31/2020 |
| Ontario Ministry of Natural Resources and Forestry | <https://www.ontario.ca/page/ministry-natural-resources-and-forestry> | 8/31/2020 |
| Quebec Ministry of Forests, Wildlife and Parks | <https://www.quebec.ca/en/government/ministere/forets-faune-parcs/> | 8/31/2020 |
| Saskatchewan Ministry of Environment | <https://www.saskatchewan.ca/government/government-structure/ministries/environment> | 8/31/2020 |
| Yukon Department of Environment | <https://yukon.ca/en/department-environment> | 8/26/2020 |
| ***United States*** | | |
| Alabama Department of Conservation and Natural Resources | <https://www.outdooralabama.com/> | 8/28/2020 |
| Alaska Department of Fish and Game | <https://www.adfg.alaska.gov/> | 8/28/2020 |
| Arizona Game and Fish Department | <https://www.azgfd.com/> | 8/28/2020 |
| Arkansas Game and Fish Commission | <https://www.agfc.com/en/> | 8/28/2020 |
| California Department of Fish and Wildlife | <https://wildlife.ca.gov/> | 8/26/2020 |
| Colorado Division of Parks and Wildlife | <https://cpw.state.co.us/> | 8/28/2020 |
| Connecticut Department of Energy and Environmental Protection | <https://portal.ct.gov/DEEP> | 8/31/2020 |
| Delaware Department of Natural Resources and Environmental Control | <https://dnrec.alpha.delaware.gov/> | 8/31/2020 |
| District of Columbia Department of Energy and Environment | <https://doee.dc.gov/> | 8/31/2020 |
| Florida Fish and Wildlife Conservation Commission | <https://myfwc.com/> | 8/31/2020 |
| Georgia Department of Natural Resources | <https://gadnr.org/> | 8/31/2020 |
| Idaho Department of Fish and Game | <https://idfg.idaho.gov/> | 8/31/2020 |
| Illinois Department of Natural Resources | <https://www2.illinois.gov/dnr/Pages/default.aspx> | 8/31/2020 |
| Indiana Department of Natural Resources | <https://www.in.gov/dnr/> | 8/31/2020 |
| Iowa Department of Natural Resources | <https://www.iowadnr.gov/> | 8/31/2020 |
| Kansas Department of Wildlife, Parks and Tourism | <https://ksoutdoors.com/> | 8/31/2020 |
| Kentucky Department of Fish and Wildlife Resources | <https://fw.ky.gov/Pages/default.aspx> | 8/31/2020 |
| Louisiana Department of Wildlife and Fisheries | <https://www.wlf.louisiana.gov/> | 8/31/2020 |
| Maine Department of Inland Fisheries and Wildlife | <https://www.maine.gov/ifw/> | 9/1/2020 |
| Maryland Department of Natural Resources | <https://dnr.maryland.gov/Pages/default.aspx> | 8/31/2020 |
| Massachusetts Department of Fish and Game | <https://www.mass.gov/orgs/department-of-fish-and-game> | 8/31/2020 |
| Michigan Department of Natural Resources | <https://www.michigan.gov/dnr/> | 8/31/2020 |
| Minnesota Department of Natural Resources | <https://www.dnr.state.mn.us/> | 8/31/2020 |
| Mississippi Department of Wildlife, Fisheries, and Parks | <https://www.mdwfp.com/> | 8/31/2020 |
| Missouri Department of Conservation | <https://mdc.mo.gov/> | 8/31/2020 |
| Montana Department of Fish, Wildlife & Parks | <http://fwp.mt.gov/> | 9/1/2020 |
| Nebraska Game and Parks Commission | <http://outdoornebraska.gov/> | 9/1/2020 |
| Nevada Department of Wildlife | <http://www.ndow.org/> | 9/1/2020 |
| New Hampshire Fish and Game Department | <https://www.wildlife.state.nh.us/> | 9/1/2020 |
| New Jersey Division of Fish and Wildlife | <https://www.njfishandwildlife.com/> | 9/1/2020 |
| New Mexico Game and Fish Department | <http://www.wildlife.state.nm.us/> | 9/2/2020 |
| New York State Department of Environmental Conservation | <https://www.dec.ny.gov/> | 9/2/2020 |
| North Carolina Wildlife Resources Commission | <https://www.ncwildlife.org/> | 9/1/2020 |
| North Dakota Game and Fish Department | <https://gf.nd.gov/> | 9/1/2020 |
| Ohio Department of Natural Resources | <https://ohiodnr.gov/wps/portal/gov/odnr/> | 9/1/2020 |
| Oklahoma Department of Wildlife Conservation | <https://www.wildlifedepartment.com/> | 9/1/2020 |
| Oregon Department of Fish and Wildlife | <https://www.dfw.state.or.us/> | 9/2/2020 |
| Pennsylvania Game Commission | <https://www.pgc.pa.gov/Pages/default.aspx> | 9/2/2020 |
| Rhode Island Department of Environmental Management | <http://www.dem.ri.gov/> | 9/2/2020 |
| South Carolina Department of Natural Resources | <https://www.dnr.sc.gov/> | 9/2/2020 |
| South Dakota Game, Fish and Parks Department | <https://gfp.sd.gov/> | 9/2/2020 |
| Tennessee Wildlife Resources Agency | <https://www.tn.gov/twra.html> | 9/2/2020 |
| Texas Parks and Wildlife Department | <https://tpwd.texas.gov/> | 9/3/2020 |
| Utah Division of Wildlife Resources | <https://wildlife.utah.gov/> | 9/3/2020 |
| Vermont Department of Fish and Wildlife | <https://vtfishandwildlife.com/> | 9/3/2020 |
| Virginia Department of Wildlife Resources | <https://dwr.virginia.gov/> | 9/3/2020 |
| Washington Department of Fish and Wildlife | <https://wdfw.wa.gov/> | 9/2/2020 |
| West Virginia Division of Natural Resources | <https://www.wvdnr.gov/> | 9/2/2020 |
| Wisconsin Department of Natural Resources | <https://dnr.wisconsin.gov/> | 9/2/2020 |
| Wyoming Game and Fish Department | <https://wgfd.wyo.gov/> | 9/2/2020 |
