# Supplementary figures and images for: Existing evidence on the effects of climate variability and climate change on ungulates in North America: a systematic map
Source: Environ Evid. 2024 Apr 4;13:8. doi: 10.1186/s13750-024-00331-8 (PMC11378825; doi:10.1186/s13750-024-00331-8)

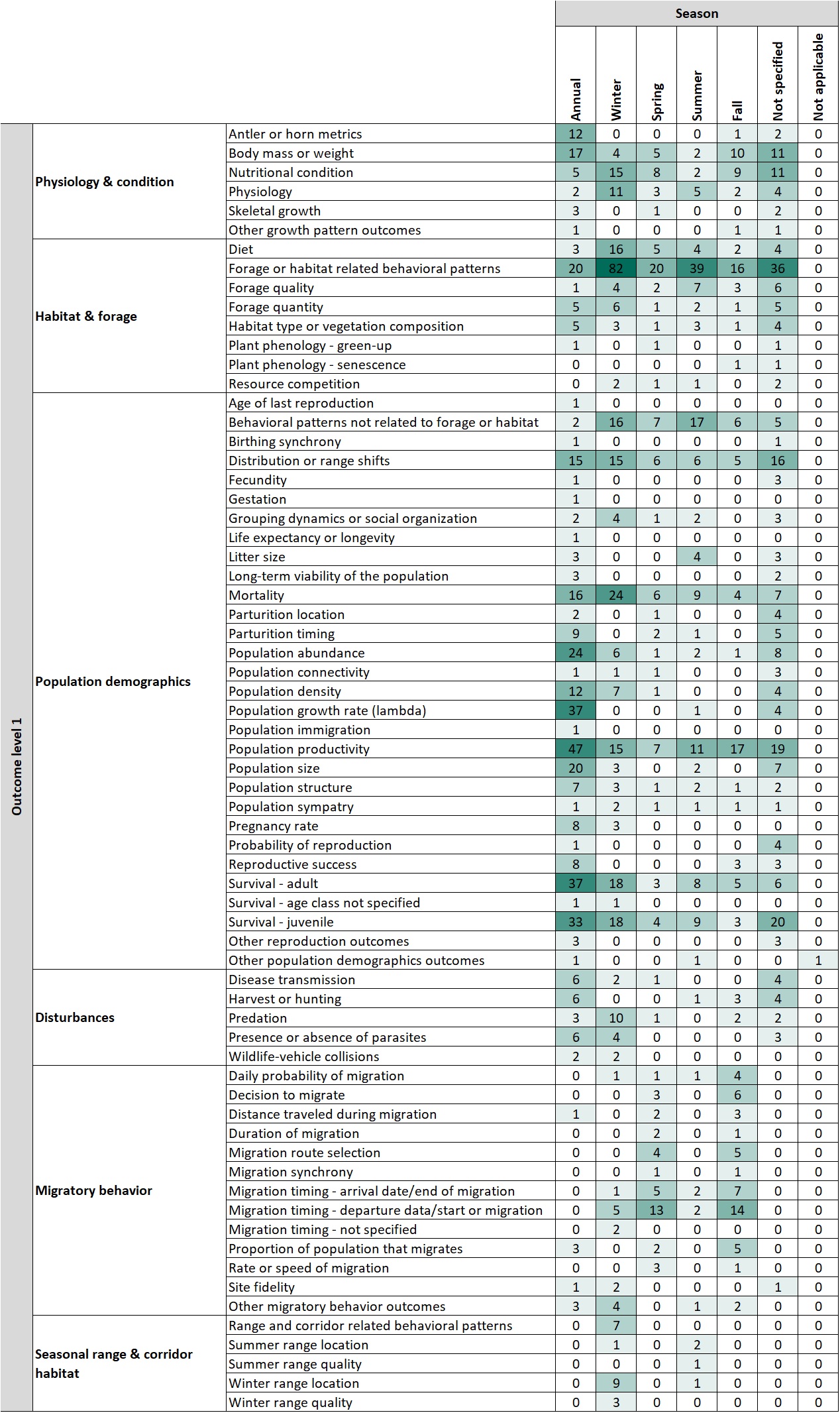

Supplement: Supplementary file 10 — Additional file 10. Distribution of articles with at least one study among outcome variables, split by seasons for which outcomes were measured. [file 13750_2024_331_MOESM10_ESM.jpg]

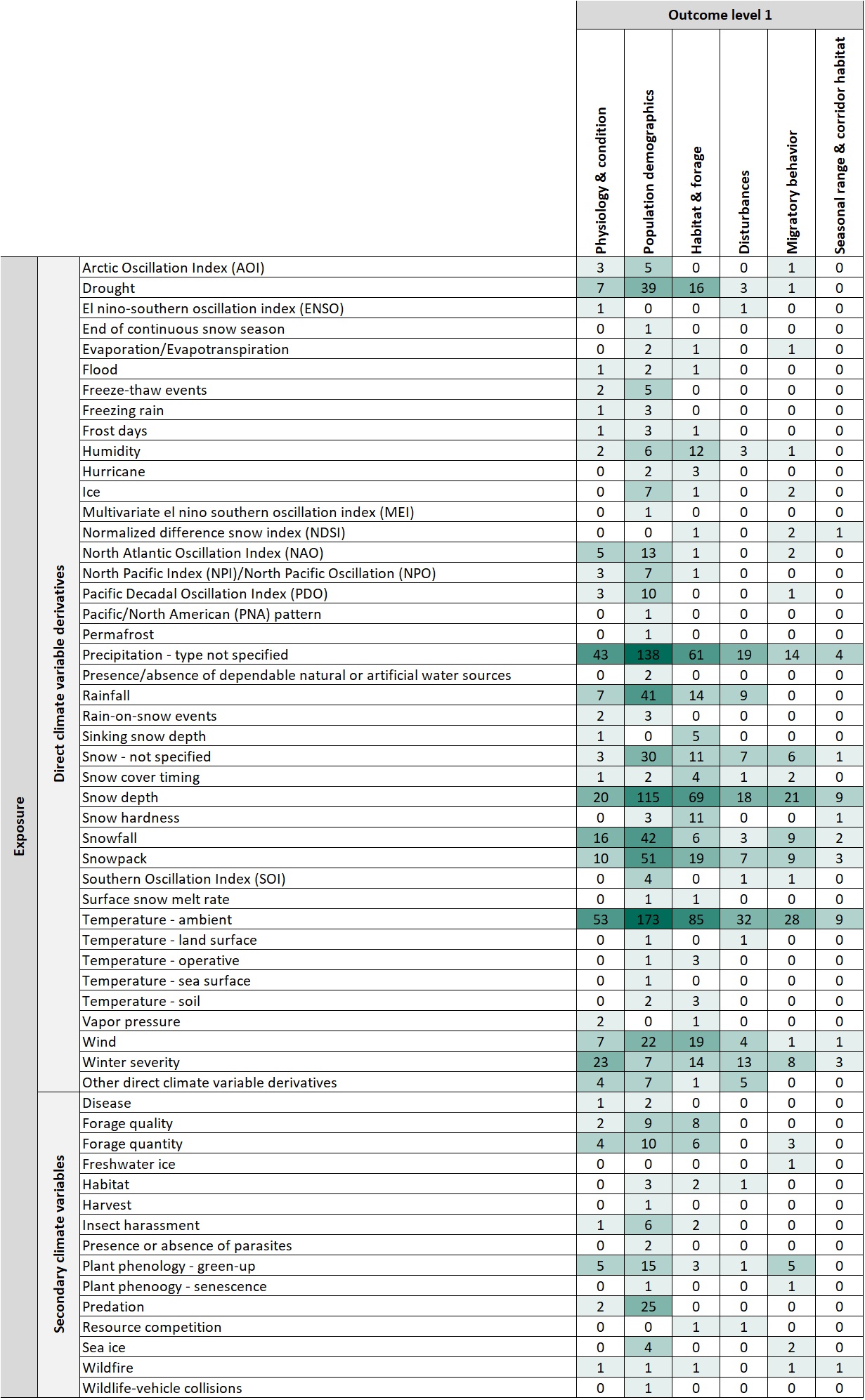

Supplement: Supplementary file 11 — Additional file 11. Distribution of articles with at least one study among level-1 outcome and exposure variables. [file 13750_2024_331_MOESM11_ESM.jpg]
